# T cell derived HIV-1 is present in the CSF in the face of suppressive antiretroviral therapy

**DOI:** 10.1371/journal.ppat.1009871

**Published:** 2021-09-23

**Authors:** Gila Lustig, Sandile Cele, Farina Karim, Anne Derache, Abigail Ngoepe, Khadija Khan, Bernadett I. Gosnell, Mahomed-Yunus S. Moosa, Ntombi Ntshuba, Suzaan Marais, Prakash M. Jeena, Katya Govender, John Adamson, Henrik Kløverpris, Ravindra K. Gupta, Rohen Harrichandparsad, Vinod B. Patel, Alex Sigal

**Affiliations:** 1 Centre for the AIDS Programme of Research in South Africa, Durban, South Africa; 2 Africa Health Research Institute, Durban, South Africa; 3 School of Laboratory Medicine and Medical Sciences, University of KwaZulu-Natal, Durban, South Africa; 4 Department of Infectious Diseases, University of KwaZulu-Natal, Durban, South Africa; 5 Department of Neurology, University of KwaZulu-Natal, Durban, South Africa; 6 Discipline of Pediatrics and Child Health, University of KwaZulu-Natal, Durban, South Africa; 7 Division of Infection and Immunity, University College London, London, United Kingdom; 8 Department of Immunology and Microbiology, University of Copenhagen, Copenhagen, Denmark; 9 Department of Medicine, University of Cambridge, Cambridge, United Kingdom; 10 Department of Neurosurgery, University of KwaZulu-Natal, Durban, South Africa; 11 Max Planck Institute for Infection Biology, Berlin, Germany; University of North Carolina at Chapel Hill, UNITED STATES

## Abstract

HIV cerebrospinal fluid (CSF) escape, where HIV is suppressed in blood but detectable in CSF, occurs when HIV persists in the CNS despite antiretroviral therapy (ART). To determine the virus producing cell type and whether lowered CSF ART levels are responsible for CSF escape, we collected blood and CSF from 156 neurosymptomatic participants from Durban, South Africa. We observed that 28% of participants with an undetectable HIV blood viral load showed CSF escape. We detected host cell surface markers on the HIV envelope to determine the cellular source of HIV in participants on the first line regimen of efavirenz, emtricitabine, and tenofovir. We confirmed CD26 as a marker which could differentiate between T cells and macrophages and microglia, and quantified CD26 levels on the virion surface, comparing the result to virus from *in vitro* infected T cells or macrophages. The measured CD26 level was consistent with the presence of T cell produced virus. We found no significant differences in ART concentrations between CSF escape and fully suppressed individuals in CSF or blood, and did not observe a clear association with drug resistance mutations in CSF virus which would allow HIV to replicate. Hence, CSF HIV in the face of ART may at least partly originate in CD4+ T cell populations.

## Author summary

The brain may be a site where HIV persists despite antiretroviral therapy (ART). Persistence can manifest as cerebrospinal fluid (CSF) escape, where HIV is detectable in the CSF but not the blood in some individuals. The reasons for CSF escape are incompletely understood. Evidence from individuals mostly on second line protease inhibitor-based ART indicates that detectable HIV in this compartment may have acquired drug resistance. In this work we investigated HIV in blood and CSF of 156 participants from Durban, South Africa. We observed a very high prevalence of CSF escape of 28%. We aimed to find the cell type responsible for producing HIV in CSF escape and whether replication occurred because of lower CSF drug levels or because the virus has developed resistance to therapy. We found that at least some of the CSF HIV was produced by T cells, and that drug resistance was not always present. This suggests that at least part of the CSF HIV reservoir may be generated by either an infection mode not requiring drug resistance for viral replication, or by latently infected CD4+ T cells trafficking to and releasing HIV in the CSF without extensive viral replication taking place.

## Introduction

HIV persistence in the face of ART necessitates lifelong adherence to treatment. The CNS may serve as one reservoir for HIV persistence [1]. HIV infection in the CNS in the absence of suppressive ART may lead to HIV-associated neurocognitive disorders (HAND). Yet, even in the presence of ART mediated suppression, sub-clinical cognitive impairment is common [2–7] and there is widespread immune activation and inflammation in the CNS [8–10]. Consistent with a role for the CNS as an HIV reservoir, a subset of individuals show CSF escape, where HIV is detectable in the CSF while being successfully suppressed below the level of detection in the blood [11–16].

Potential reasons for a CNS reservoir include reduced drug levels. Drug levels of efavirenz (EFV), emtricitabine (FTC), and tenofovir (TFV) in the CSF are reduced approximately 200-fold, 2-fold, and 20-fold, respectively in the CSF relative to blood [17–19]. Since the majority of individuals do not show detectable virus in the CSF, these lowered ART levels seem to be sufficient to suppress viremia. However, it is unclear if ART levels in the CSF are lower in individuals with neurosymptomatic CSF escape in South Africa, accounting for the lack of effective suppression in this compartment and possibly evolution of drug resistance mutations. Mutations could include the M184V or M184I resistance mutation to FTC, a drug which would provide selective pressure since it has good penetration to the CNS [14, 20–24].

There is evidence for compartmentalized HIV infection in the CNS, indicating that CNS specific cell subtypes may be involved [25–27]. HIV infected, long lived CNS resident host cells such as microglia and perivascular macrophages may be responsible for the HIV reservoir in the CNS [11, 27–31]. HIV infection may not be appreciably cytotoxic in these cells [32] and these cells are resistant to cytotoxic T lymphocyte killing [33], allowing long-term infected cell persistence without new cycles of re-infection.

T cells are also present in the CNS. CSF contains trafficking T cells, mostly CD4+ memory cells, which enter across the choroid plexus [34]. T cell-tropic HIV is present in the CSF in some individuals [27, 28, 35] and CSF HIV was found to have fast decay kinetics upon ART initiation, consistent with the short half-lives of infected T cells [36].

Here we aimed to determine the cellular source of HIV in the brain and whether lower ART levels relative to fully suppressed individuals account for CSF escape in individuals from Durban, South Africa. This is the first time in our knowledge where CSF escape in Sub-Saharan Africa has been investigated in a relatively large number of participants on ART [37]. Since accessing HIV infected cells from the CNS is challenging, we chose a method which could determine the cell-of-origin of cell-free HIV sampled from the CSF. Upon viral budding, the HIV envelope contains host surface markers [38, 39] since HIV uses the cellular plasma membrane as its envelope. The host surface markers can be bound with antibodies and detected using a variety of techniques [40–42] including electron microscopy [39], mass spectrometry [43], flow cytometry [44, 45], and immunomagnetic capture [46–50]. Many of these studies report that HIV derived from macrophage lineage cells expresses CD36 [43–46, 48–50], a scavenger receptor [51–56], on its envelope. HIV derived from T cells expresses CD26 [44, 46, 48–50], a dipeptidyl-peptidase involved in T cell activation [57]. We tested the ability of CD26 and CD36 to differentiate between T cells and other cell types. We found that in samples obtained from the South African study participants, CD26 was T cell specific. CSF escape HIV had CD26 on its surface, consistent with at least partial T cell origin of CSF escape virus. We also observed that CSF ART concentrations in CSF escape were not significantly different from those of participants with viral suppression and did not detect a clear association with drug resistance mutations, indicating that detectable T cell origin HIV can persist in the CSF despite suppressive ART.

## Materials and methods

### Ethical statement

CSF and matched blood were obtained from participants indicated for lumbar puncture enrolled at Inkosi Albert Luthuli Central Hospital and King Edward VIII Hospital in Durban, South Africa after written informed consent (University of KwaZulu-Natal Institutional Review Board approval BE385/13). Discarded tissue from the field of neurosurgery was obtained from two participants enrolled at Inkosi Albert Luthuli Central Hospital indicated for neurosurgery after written informed consent (University of KwaZulu-Natal Institutional Review Board approval BE315/18). Blood for PBMC, CD4+ and CD14+ cell isolation was obtained from adult healthy volunteers after written informed consent (University of KwaZulu-Natal Institutional Review Board approval BE022/13 and BE083/18). Lymph nodes were obtained from the field of surgery of participants undergoing surgery for diagnostic purposes and/or complications of inflammatory lung disease. Informed consent was obtained from each participant, and the study protocol approved by the University of KwaZulu-Natal Institutional Review Board (approval BE024/09).

### Statistical tests

Data is described with the non-parametric measures of median and interquartile range, and significance determined using the non-parametric Mann-Whitney U test for pairwise comparisons, Fisher exact test for pairwise comparisons of frequencies, and the Kruskal-Wallis test with multiple comparison correction by the Dunn Method for comparisons involved more than two populations. All tests were performed using Graphpad Prism 8 software.

### CSF sample collection and processing

Fresh CSF and matching blood samples were transported to the laboratory and processed immediately. Two separate EDTA tubes of 4 mL blood were sent for testing in parallel: one for a CD4/CD8 count at an accredited diagnostic laboratory (Ampath, Durban, South Africa) and one for viral load at an accredited diagnostic laboratory (Molecular Diagnostic Services, Durban, South Africa, using the RealTime HIV-1 viral load test on an Abbott machine). CSF samples were spun for 10 min at 1000 g to remove debris. CSF supernatant was frozen in 1 mL aliquots at -80°C. One aliquot of 100 *μ*l CSF was sent for viral load (Molecular Diagnostic Services) directly after the spin. Plasma was processed by spinning the whole blood for 10 min at 1300 g, no brake. The top layer was removed and stored in 1 mL aliquots at -80°C.

### Antiretrovirals, viruses and cells

The following reagents were obtained through the AIDS Research and Reference Reagent Program, National Institute of Allergy and Infectious Diseases, National Institutes of Health: the antiretrovirals EFV, FTC, and TFV and the plasmid for the macrophage tropic pNL4–3(AD8) HIV molecular clone. NL4–3 and NL4–3(AD8) HIV stocks were produced by transfection of HEK293 cells with the molecular clone plasmids using TransIT-LT1 (Mirus) transfection reagent. Supernatant containing released virus was harvested two days post-transfection and filtered through a 0.45 micron filter (GVS) and stored in 0.5 mL aliqouts at -80°C. The number of HIV RNA genomes in viral stocks was determined using the RealTime HIV-1 viral load test (Molecular Diagnostic Services, Durban, South Africa). RevCEM-E7 cells were generated as previously described [58]. Cell culture medium was complete RPMI 1640 supplemented with L-glutamine, sodium pyruvate, HEPES, non-essential amino acids (Lonza), and 10% heat-inactivated FBS (Hyclone). PBMCs were isolated by density gradient centrifugation using Histopaque 1077 (Sigma). CD4+ or CD14+ cells were positively selected using either CD4 or CD14 Microbeads loaded onto MACS separation columns according to manufacturer’s instructions (Miltenyi Biotec). CD4+ PBMCs were grown in the above cell media supplemented with 5 ng/mL IL-2 (Peprotech) and 10 *μ*g/mL PHA (Sigma-Aldrich). Monocyte-derived macrophages were grown in RPMI 1640 supplemented with 10% human serum (Sigma) with added L-glutamine, sodium pyruvate, HEPES, and non-essential amino acids (Lonza), and differentiated with 20 ng/mL M-CSF (Peprotech) for 10 days.

### Surface staining and detection of CD26 and CD36 markers by flow cytometry

Staining of MDM and PBMC T cells: MDM and CD4+ PBMCs were generated as described above. PBMCs were washed once in PBS-/-. MDM were washed once in PBS-/- then incubated in 5 mM EDTA in PBS-/- for 30 minutes on ice. Macrophages were collected by pipetting vigorously and the remaining attached cells were removed by gentle scraping. Cells were then incubated with either CD3-APC and CD8-Bv500 (PBMC) or CD68-APC (MDM) and CD26-FITC and CD36-PE (Biolegend) fluorescently labelled antibodies in staining buffer (PBS-/- with 3%FCS) for 30 min on ice. The samples were then washed, resuspended in 400 *μ*L staining buffer and acquired on a FACSCalibur machine (BD Biosciences). Results were analyzed using FlowJo software. Staining of LN cells: LN from the field of indicated cardiothoracic surgery were cellularized by gentle mechanical dissociation and cryopreserved. For staining, LN cells were thawed, washed once in PBS-/-, then incubated with the following fluorescently conjugated antibody mix for 30 min: CD45-HV500, CD19-BV785, CD3-PE-CF594, CD4-AF700 HLA-DR-BV605 (all BD Biosciences), CD26-FITC, CD36-PE (Biolegend), and the LIVE/DEAD Fixable Near-IR Dead Cell Stain (ThermoFisher Scientific). Cells were then fixed and permeabilized using the BD Cytofix/Cytoperm Fixation/Permeabilization kit (BD Biosciences) according to the manufacturer’s instructions. Cells were then stained with CD68-APC antibodies (Biolegend). Cells were washed, fixed in 2% formaldehyde and acquired on a BD ARIA Fusion flow cytometer (BD Biosciences).

### Isolation and surface staining of human microglia

Meninges discarded tissue samples were transported immediately to the laboratory for processing. The tissue was dissociated (Brain Dissociation kit, Miltenyi). The dissociated cells underwent myelin removal (Myelin removal kit, Miltenyi) and CD11b+ cells were further purified (CD11b microglia Microbeads, Miltenyi). All kits were used according to the manufacturers instructions. The cells were then surface stained with fluorescently conjugated antibodies for microglial markers: CD11b-APC, CD45-Bv605 and P2RY12-Bv421, CD26-FITC, and CD36-PE (Biolegend). The cells were incubated with antibodies for 30 min on ice, washed, resuspended in 500 *μ*L staining buffer, and acquired on a FACS Fortessa (BD Biosciences). Results were analyzed using FlowJo software.

### *in vitro* generation of virus from PBMC or MDM

To generate PBMC origin HIV, PBMCs isolated and activated as described above were infected with 2 × 10^6^ RNA copies/mL NL4–3(AD8) for 24 hours. Cells were then washed 4 times in growth medium to remove the input viral stock and incubated for 4 days (approximately two full virus cycles) for a total infection time of 5 days. Virus containing supernatant was collected, centrifuged at 300 g for 5 minutes then filtered through a 0.45 micron syringe filter (GVS) to remove cells and cellular debris. The number of virus genomes in the virus stock was determined using the RealTime HIV-1 viral load test (Molecular Diagnostic Services). To generate monocyte-derived macrophage virus, CD14+ monocytes were isolated and differentiated as described above. Cells were then infected with with 2 × 10^7^ RNA copies/mL NL4–3(AD8) for 24 hours. Cells were washed 6 times with RPMI to remove input virus, and growth media was replaced. Half the volume of media was replaced every 3 days for 14 days for a total infection time of 15 days. Virus containing supernatant was collected, centrifuged and filtered, and viral load determined as for PBMC virus. We used 7 different donors blood donors: 6009, 6013, 6017, 6019, 6026, 6033 and 6049.

### Host cell marker detection on virion surface

The following protocol was adapted from the *μ*MACS Streptavidin Kit protocol (Miltenyi): 1 *μ*g of biotinylated antibodies to CD26 or CD36 (Ancell) were added to 1 mL of virus, mixed and incubated for 30 min at room temperature. Next, 30 *μ*L of strepavidin MicroBeads (Miltenyi) were added per sample, mixed and incubated at room temperature for 10 min. The samples were then loaded onto a *μ*Column, washed three times and bound virus eluted. Clinical virus samples were centrifuged for 13,000 g for 30 seconds to clear debris before addition of antibodies. To avoid overloading columns, *in vitro* generated virus stocks from either PBMCs or macrophages were diluted to approximately 10^4^ RNA copies/mL in PBS before incubation with antibodies. We used the 7 donors above. Virus was precipitated once for six of the donors, whereas 6049 was done in duplicate. The number of virus genomes in elutions from *μ*Columns was determined using the RealTime HIV-1 viral load test (Molecular Diagnostic Services). Out of 22 CSF escape samples, 11 had sufficient volume for the assay (2 mL). One sample which showed neither detectable CD26 nor CD36 was excluded from the analysis due to possible degradation of the virus.

### Generation of YFP-NL4–3(AD8)

pNL4–3(AD8) was used as the source of the macrophage tropic HIV ENV which was excised from pNL4–3(AD8) using BamHI and EcoRI restriction enzymes (NEB) and ligated using T4 ligase (Invitrogen) into the pNL4–3-YFP vector (gift from David Levy), replacing the NL4–3 X4 specific HIV envelope gene between the unique EcoRI-BamHI restriction sites.

### Detection of ART concentrations in CSF and matched plasma by LC-MS/MS

Sample analysis was performed using an Agilent High Pressure Liquid Chromatography (HPLC) system coupled to the AB Sciex 5500, triple quadrupole mass spectrometer equipped with an electrospray ionization (ESI) TurboIonSpray source. The LC-MS/MS method was developed and optimised for the quantitation of tenofovir, emtricitabine, efavirenz, lopinavir, ritonavir, nevirapine, zidovudine, lamivudine, abacavir, atazanvir and dolutegravir in the same sample. A protein precipitation extraction method using acetonitrile was used to process clinical plasma and CSF samples. The procedure was performed using 50 *μ*L of plasma or CSF. 50 *μ*L of water and 50 *μ*L of ISTD solution was added and the sample was briefly mixed. 150 *μ*L of acetonitrile was subsequently added to facilitate protein precipitation, vortex mixed and centrifuged at 16000 g for 10 min at 4°C. 170 *μ*L of the clear supernatant was then transferred to a clean micro-centrifuge tube and dried down using a SpeedVac dryer set at 40°C. The dried samples were then reconstituted in 100 *μ*L of 0.02% sodium deoxycholate (Sigma) in Millipore filtered water, vortex mixed, briefly centrifuged, placed in a small insert vial, capped, placed in the auto sampler compartment (maintained at 4°C) and analyzed using LC-MS/MS. The analytes were separated on an Agilent Zorbax Eclipse Plus C18 HPLC column using gradient elution. The column oven was set at 40°C, a sample volume of 2 *μ*L was injected and the flow rate was set to 0.2 mL/min. Mobile phase A consisted of water with 0.1% formic acid and B consisted of acetonitrile with 0.1% formic acid. The drug analytes were monitored using multiple-reaction monitoring mode for positive ions except for efavirenz which was monitored in the negative ion scan mode. Analyst software, version 1.6.2 was used for quantitative data analysis. Blanked values for EFV, FTC and TFV were in the range of 3 ng/mL and this was set as the detection limit.

## Results

### Prevalence of CSF escape

We sampled CSF and matched blood from participants living with HIV (*n* = 156) clinically indicated for lumbar puncture as part of their diagnostic workup in Durban, South Africa (Table 1). The majority (*n* = 80 or 51%) of participants showed an undetectable HIV viral load (VL) in the blood. Out of the subgroup of successfully ART suppressed individuals in the blood, 22 (28% of those with an undetectable VL in the blood) had detectable viremia in the CSF (CSF escape). The remainder of the participants (*n* = 76 or 49% of the total) had detectable blood viremia either because they were treatment naïve (*n* = 34 or 45% of the viremic group) or failing ART (*n* = 42 or 55% of the viremic group). We note that the CSF escape detected in this study is likely neurosymptomatic CSF escape, leading to clinically indicated lumbar puncture (see S1 Table for indications). This form of CSF escape may have a different cellular source and infection dynamics relative to asymptomatic CSF escape [59].

**Table 1:** Participant details.

Infection state	n (F/M)	Age (IQR)	VL CSF (IQR)	VL Plasma (IQR)	CD4 count (IQR)	Years ART (IQR)	Fraction co-infect	Fraction pleocytosis^1^
CSF escape	22	40	2720	< 40	474	4.8	0.27	0.45
	(11/11)	(34–46)	(897–28380)		(344–585)	(1.3–7.2)		
Suppressed	58	34	< 40	< 40	509	2.3	0.33	0.33
	(37/21)	(29–42)			(193–724)	(0.9–6.1)		
Viremic (total)	76	33	3680	14956	246	1.0^2^	0.38	0.41
	(48/28)	(28–40)	(173–26210)	(699–70672)	(91–411)	(0.08–5)		
Viremic (naïve)	34	35	9819	44902	324	N/A	0.32	0.53
	(20/14)	(30–44)	(3255–78440)	(13935–240120)	(104–493)			
Viremic (ART)	42	32	1280	1950	219	1.0	0.43	0.31
	(28/14)	(27–36)	(< 40–5624)	(245–31754)	(52–379)	(0.08–5)		

VL in HIV RNA copies/mL blood. Limit of detection 40 copies/mL.

^1^Defined as >5 leukocytes per *μ*L of CSF.

^2^Of viremic participants on ART.

The CSF escape group was significantly older compared to the suppressed group (*p* = 0.018, Mann-Whitney U test). Otherwise, the CSF escape group was very similar to the suppressed group, except for the VL in the CSF. The groups did not differ in either median CD4 count (*p* = 0.78, Mann-Whitney), years on ART (*p* = 0.25, Mann-Whitney), fraction of individuals with diagnosed co-infections (*p* = 0.78, Fisher’s exact test) or the ratio between males and females (*p* = 0.79, Fisher’s exact test). Pleocytosis, abnormally high white blood cell concentrations in the CSF usually indicating co-infection with another pathogen in the CNS, occurred in 45% of CSF escape participants and 33% of suppressed participants. However, the trend of higher pleocytosis in CSF escape was not statistically significant (*p* = 0.31, Fisher’s exact test). Surprisingly, median CSF VL in CSF escape participants was not significantly different from that of viremic participants (*p* = 0.96, Mann-Whitney). However, when compared to the treatment naïve and ART experienced viremic participant subgroups separately, the CSF escape participants showed a significantly lower median CSF VL compared to viremic naïve (*p* = 0.022, Mann-Whitney) and significantly higher VL compared to viremic participants on ART (*p* = 0.039, Mann-Whitney).

To exclude the confounding effect of treatment regimen [16], we concentrated on samples from suppressed or CSF escape participants on a regimen of EFV, FTC, and TFV (first line therapy at the time of sample collection), and on participants where sufficient sample volume was available, for subsequent analysis.

### CD26 expression differentiates between T cells and macrophages and microglia

**Fig 1:**
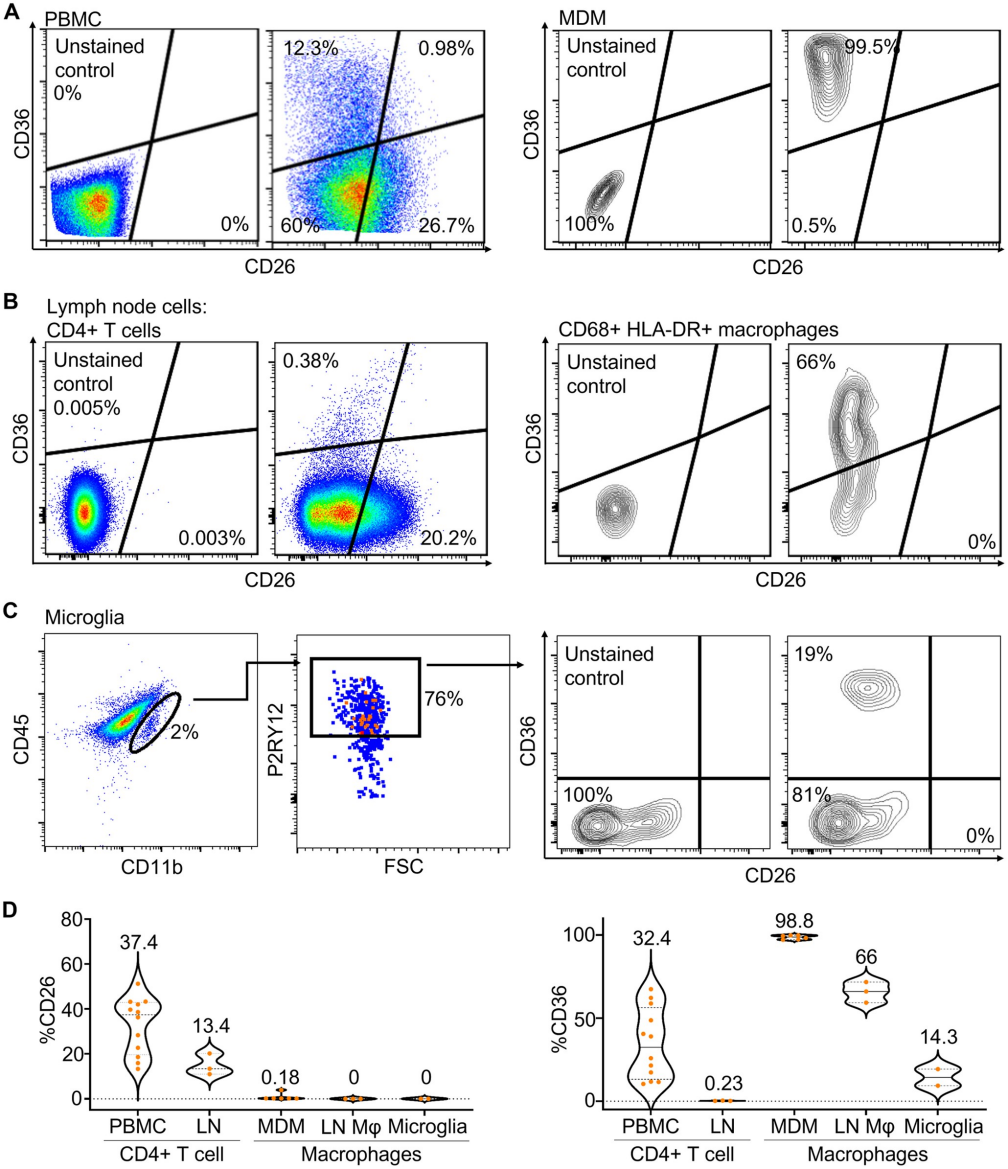
Expression of CD26 and CD36 surface markers on T cells and macrophages. (A) Flow cytometry detection of CD36 and CD26 on the surface of CD3+ gated PBMC (left) or CD68+ monocyte derived macrophages (MDM) (right). (B) CD26 and CD36 detection on lymph node (LN) origin live CD45+CD19-CD3+CD4+ T cells (left) or live CD45+CD3-CD19-CD68+HLA-DR+ macrophages (right). Staining was performed on three LN from the field of indicated surgery of study participants 024–09-0233, 024–09-0274, and 024–09-0257. Shown is a representative result (participant 024–09-0257). (C) primary human CD11bmidP2RY12+ microglial cells. (D) Violin plots of the fraction of cells expressing CD26 (left) and CD36 (right) of CD4 T cells from PBMC (n = 12) or LN (n = 3), MDM (n = 6), lymph node macrophages (LN M*ϕ*, n = 3)), or microglia (n = 2). Number above each plot indicates median percent positive cells.

Previous work detecting host cell markers on the virion surface [46–50] converged on CD26 (T cell) and CD36 (macrophage lineage) host cell markers on the surface of the HIV virion as the most robust markers for differentiation between cell types using this approach. We therefore proceeded to test whether these markers could indeed differentiate T cells from macrophages and microglia in our study population (Fig 1). We detected CD26 and CD36 on: 1) CD3+ T cells from peripheral blood mononuclear cells (PBMC); 2) CD68+ monocyte derived macrophages (MDM) (Fig 1A, see S1 Fig for gating strategy); 3) CD4+ T cells from lymph nodes (LN); 4) CD68+HLA-DR+ macrophages from LN (Fig 1B, see S1 Fig for gating strategy for LN cells); 5) primary human CD11b+P2RY12+ microglia from discarded neurosurgical tissue (Fig 1C).

The level of CD26 showed a clear demarcation between T cells in the two compartments and macrophages and microglia (Fig 1D, left panel). While there were differences in CD26 expression between T cells in PBMC and LN (37% versus 13% positive cells), MDM, LN macrophages, and microglia had negligible or undetectable CD26 levels. In contrast, it was less clear that CD36 could differentiate cell types in the South African population studied here (Fig 1D, right panel). While LN T cell showed negligible levels, CD36 expression in PBMC in our study population was substantial and within the range of expression in macrophages and microglia (Fig 1D, right panel). Since only about one quarter of T cells are positive for CD26, cells that are negative for CD26 can either be T cells or macrophages/microglia. However, CD26 positives cells are not macrophages/microglia.

Given that CD36 in our assays could not discriminate well between T cells and macrophages/microglia, we have focused our analysis on CD26 level as an indicator of T cell origin.

### Host cell markers on the virion surface are consistent with presence of T cell origin HIV

**Fig 2:**
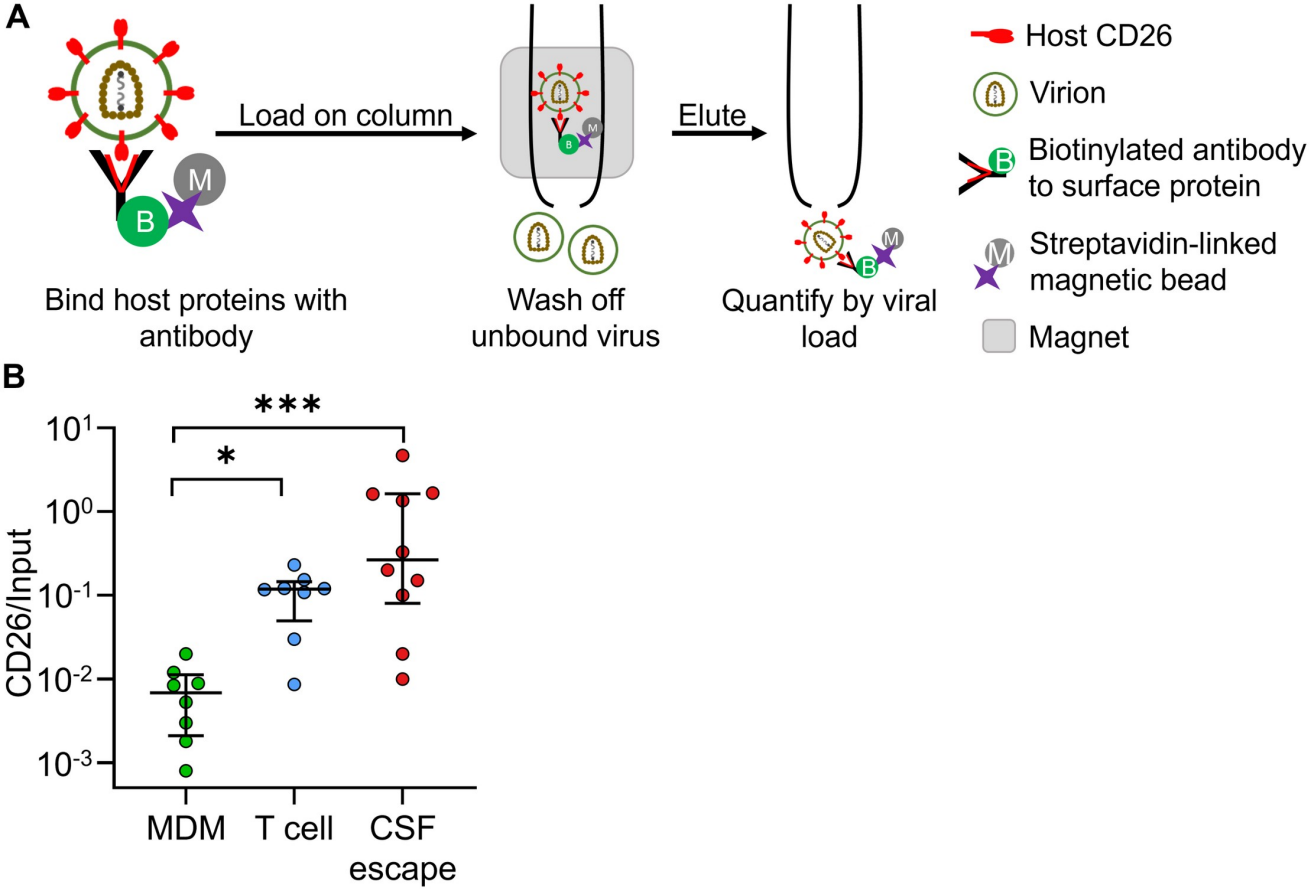
HIV from CSF escape contains the CD26 host surface marker consistent with T cell origin. (A) Schematic of method. Cell-free virus was bound to columns with anti-CD26 antibodies linked to magnetic beads. After washing off unbound virus, virus bound to the columns was eluted and quantified using a viral load assay. (B) Monocyte derived macrophages or peripheral blood mononuclear cells were infected with NL4–3(AD8) macrophage tropic HIV and supernatants from infected cells were loaded on columns with CD26 antibody and normalized by viral input. Results were compared with virus derived from study participants with CSF escape. Shown are median and IQR of supernatants derived from 10 CSF escape participants or *in vitro* infections of PBMC or MDM from 7 healthy blood donors, where blood from one of the donors was used twice (n = 8 total experiments). p-values are * < 0.05; *** < 0.001 as determined by Kruskal-Wallis test with Dunn multiple comparison correction.

To detect cellular origin of CSF HIV using CD26, we measured the ratio of the number of virions expressing CD26 on their surface to the total number of virions in the sample. Both quantities were measured using a viral load (VL) assay as follows. We coupled CD26 antibodies to magnetic beads and added the sample containing either virus from CSF or plasma samples, or *in vitro* produced virus to a column containing the bound antibodies. We performed a VL assay to determine: 1) the number of CD26 antibody bound virions after washing off unbound virions, and 2) the VL of the virus suspension added to the column (Fig 2A). We normalized by the VL of the virus added to the columns so that the measurement would not be affected by the absolute VL, which varied between study participants. We infected either monocyte derived macrophages (MDM) or peripheral blood mononuclear cells (PBMC) with the macrophage tropic HIV strain NL4–3(AD8), which infects both CD4 T cells and macrophages, to test whether CD26 level can differentiate between the virions produced in each cell type using this approach. Using YFP labelled macrophage tropic HIV (YFP-NL4–3(AD8)), we observed no infection of CD14+ monocytes in PBMC (S2 Fig), indicating that the PBMC derived virus originated in T cells. There was approximately an order of magnitude increase in the CD26 level normalized by input VL (*p* < 0.05, Kruskal-Wallis test with Dunn multiple comparison correction) in virus originating from PBMC relative to MDM (Fig 2B, see S3 Fig for raw VL). Similarly to what we observed using CD26 and CD36 in cell surface labelling, CD26 on the viral surface could specifically differentiate between T cells and macrophages as the viral source, but CD36 could not (Fig 2B and S3 Fig).

We next used the CD26 normalized by input VL to infer the cellular origin of *in vivo* derived CSF escape virus for CSF samples with sufficient volume (11 samples excluded due to insufficient volume (2 mL minimum) for the assay and one excluded based on poor sample quality (Materials and methods)). We compared the CD26/input ratio obtained from CSF escape HIV with the *in vitro* values for MDM and PBMC (Fig 2B). The median CSF escape CD26 level was significantly higher than the ratio obtained from HIV derived from MDM infection (p-value ***< 0.001, Kruskal-Wallis test with Dunn multiple comparison correction). In contrast, it was similar to the CD26 level in T cell derived virus from *in vitro* infected PBMC (*p* > 0.99, using the same test).

If the number of virions bound to the anti-CD26 antibodies from the CSF is very small, it may be difficult to accurately estimate the ratio of virions expressing CD26 relative to input. We therefore also examined the absolute numbers of anti-CD26 bound and input virions (S4 Fig). To ensure that the range of observed values is taken into account, we calculated the geometric mean of the data. We observed that even the CSF with the lowest number of viral copies measured was approximately an order of magnitude above the detection limit of the VL assay of 40 HIV RNA copies/mL. The geometric mean of the number of CD26 expressing virions, at about 1600 copies/mL, was approximately 4-fold lower than the total number of virions in the sample. Such a decrease would be expected even if all the virus was produced in T cells, since we observed that only about one quarter of T cells express detectable CD26 (Fig 1). Given that the number of CD26 expressing virions was neither at the assay detection limit nor a small proportion of the total virions in the sample, it is unlikely that our estimate of the ratio of CD26 expressing virions in the total virus pool is strongly affected by these factors. We therefore conclude that the measurement of CD26 on HIV from the CSF of individuals with CSF escape is consistent with the presence of T cell origin virus.

**Fig 3:**
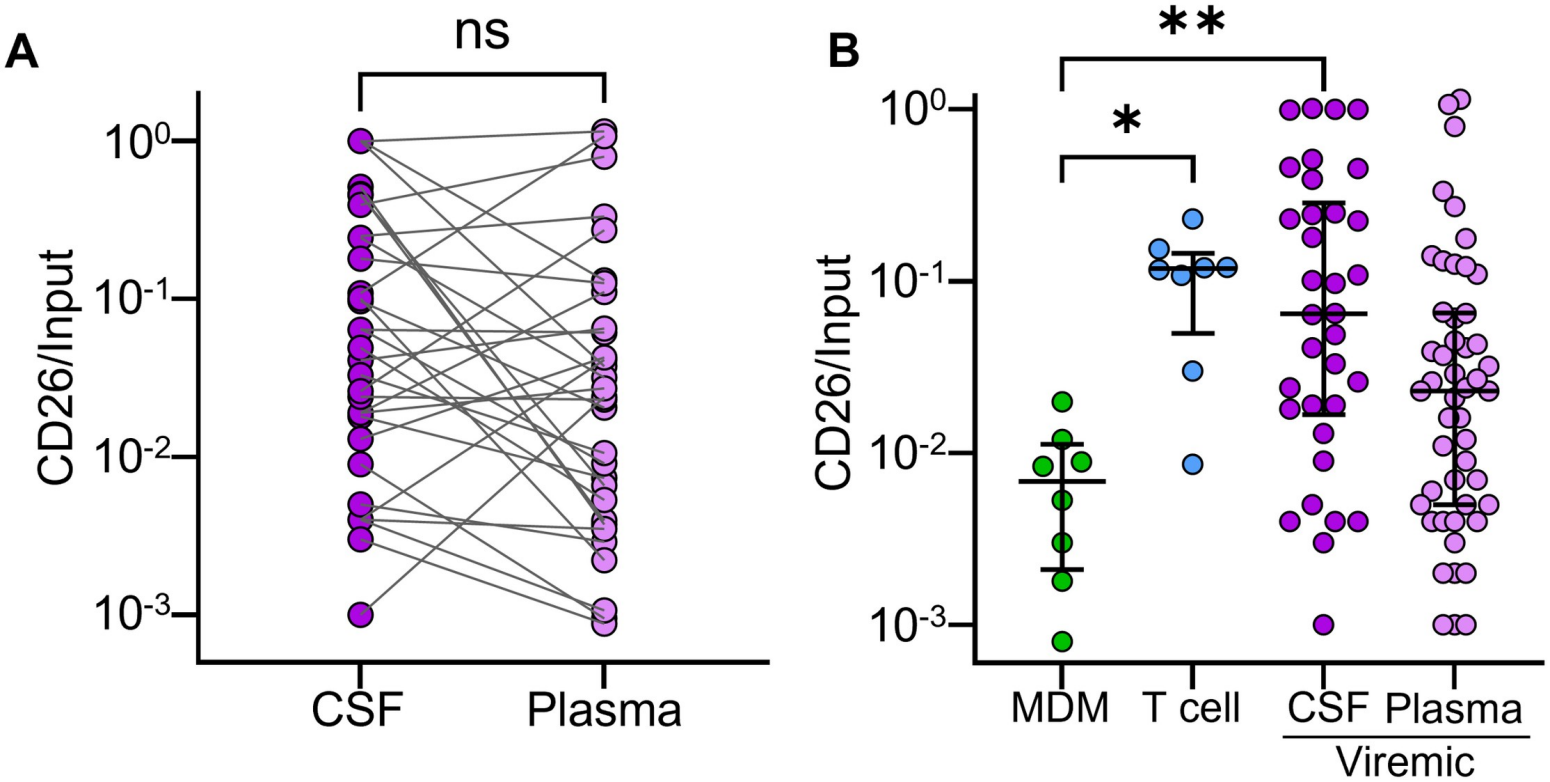
*in vivo* cellular source of HIV from viremic participants. (A) CD26/input ratio in participant matched CSF and plasma from viremic participants where both were detectable (*n* = 32). (B) CD26/input ratio for all available samples (n = 34 for CSF, n = 48 plasma) from CSF and plasma of viremic study participants compared to virus derived from *in vitro* infected MDM and T cells. p-values are * < 0.05; ** < 0.01 as determined by the Kruskal-Wallis test with Dunn multiple comparison correction.

We also investigated origin of CSF and plasma virus from plasma viremic participants (Fig 3). There was no significant trend to higher or lower CD26 level between the matched CSF and plasma compartments from the same participant (Fig 3A). The median CD26/input ratio from CSF virus of participants with viremia was significantly different from that of MDM generated virus and consistent with the presence of T cell origin virus. However, there was a wide range of CD26 levels between participants, including low CD26 levels similar to virus produced *in vitro* by MDM (Fig 3B). In plasma of viremic participants, the range of CD26 levels was large and overlapped CD26 expression from both T cell and MDM derived virus (Fig 3B).

### CSF ART levels similar between individuals with CSF escape and full suppression

**Fig 4:**
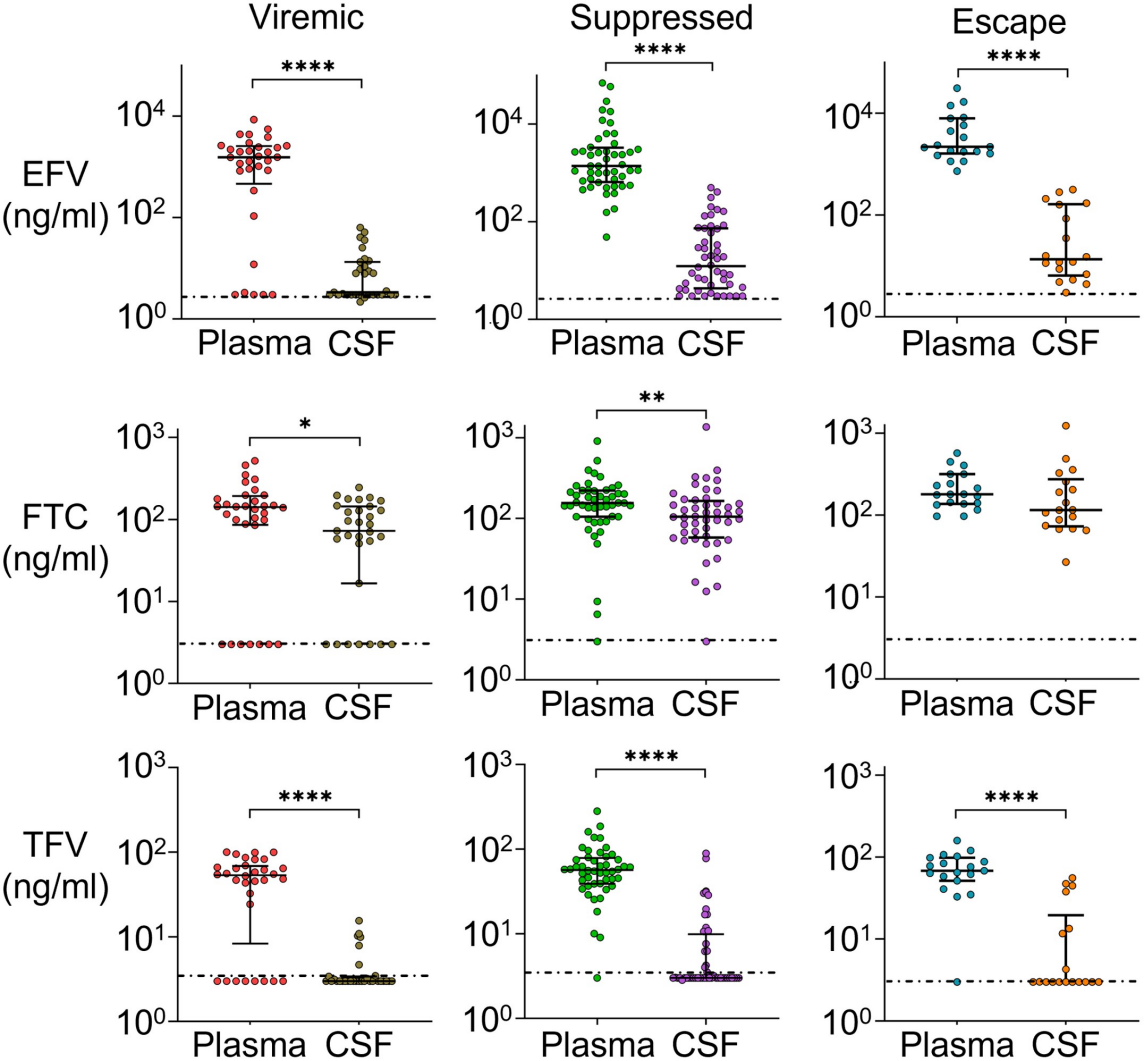
Sharp drop in EFV levels between plasma and CSF. Medians and IQR of EFV, FTC, and TFV concentrations in the plasma versus CSF in participants who were viremic on ART (plasma n = 32, CSF n = 31), suppressed (plasma and CSF n = 48), or showed CSF escape (plasma n = 19, CSF n = 18). Horizontal dotted line indicates limit of detection (3 ng/mL). p-values are: *< 0.05; **< 0.01; ****< 0.0001 using the Mann-Whitney U test.

To investigate whether CSF escape is due to lowered CSF ART levels, we measured the plasma and CSF concentrations of ART regimen components using liquid chromatography–tandem mass spectrometry (LC-MS/MS). We compared drug concentrations between viremic participants on ART, suppressed participants, and CSF escape participants. Confirming previous results [19, 60, 61], we detected a sharp drop in EFV concentrations in the CSF relative to plasma (Fig 4), with EFV about two orders of magnitude lower. The decline in FTC levels between plasma and CSF was much more attenuated, showing FTC has good penetration into the CSF. TFV levels were close to or below the limit of detection in the CSF for most participants, and about an order of magnitude higher in the plasma.

**Fig 5:**
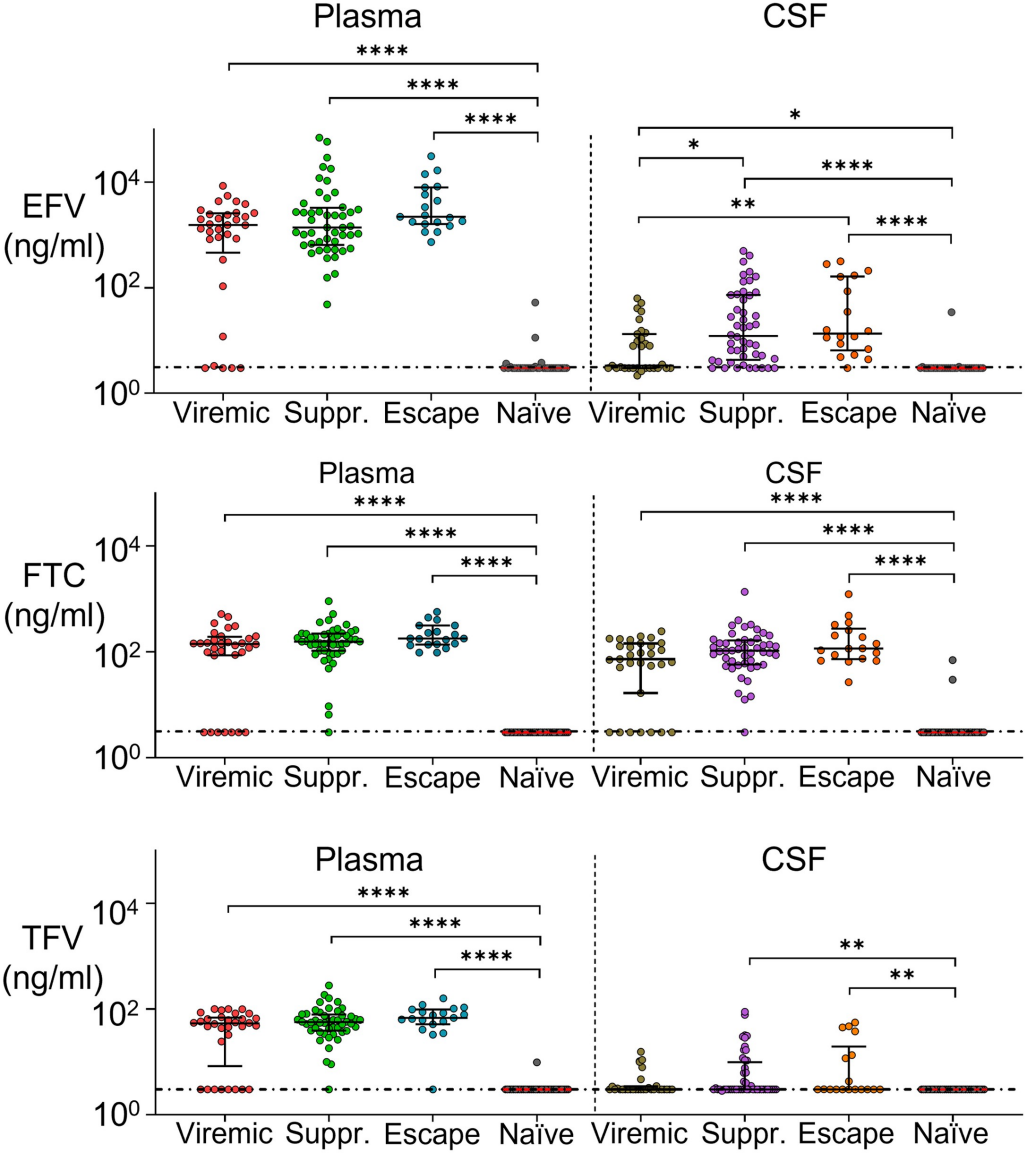
ART concentrations in plasma and CSF are similar in individuals with full suppression and CSF escape. Medians and IQR of EFV, FTC, and TFV concentrations in the plasma (left) and CSF (right) in individuals who were viremic on ART (plasma n = 32, CSF n = 31), suppressed (plasma and CSF n = 48), showed CSF escape (plasma n = 19, CSF n = 18), or were reported as treatment naïve (plasma n = 33, CSF n = 31). Horizontal dotted line indicates limit of detection (3 ng/mL). p-values are: *< 0.05; **< 0.01; ****< 0.0001 using the Kruskal-Wallis test with Dunn multiple comparison correction.

We compared drug levels between groups, and included participants reported to be treatment naïve (Fig 5). We observed that, as expected, the treatment naïve group had drug levels below the threshold of detection. Viremic participants showed a bimodal distribution of drug levels in the plasma and CSF, likely corresponding to two subgroups: individuals either failing therapy or who are non-adherent. There was no significant difference between levels of EFV between suppressed and CSF escape participants in either the plasma or CSF (*p* = 0.83 and *p* > 0.99 for plasma and CSF respectively, Kruskal-Wallis test with Dunn multiple comparison correction). The same was true of FTC (*p* > 0.99 and *p* > 0.99 for plasma and CSF respectively) and TFV (*p* > 0.99 and *p* > 0.99 respectively). This indicates that there is no reduction in CSF drug levels in CSF escape individuals relative to individuals with full suppression.

### Drug resistance mutations not essential for a detectable viral load in the CSF

One possible explanation for CSF escape is accumulation of drug resistance mutations (DRMs) from previous gaps in adherence or other factors. We therefore sequenced CSF virus. In participant samples where CSF virus sequencing was successful (Table 2), we observed DRMs including the M184V high level resistance mutation to FTC and the L100I, K103N, V106M, and G190A mutations to EFV (https://hivdb.stanford.edu/).

**Table 2:** Drug resistance mutations in CSF.

PID	VL (copies/ml) Pl/CSF	Drug resistance mutation	TFV (ng/ml) Pl/CSF	FTC (ng/ml) Pl/CSF	EFV (ng/ml) Pl/CSF	LPV (ng/ml) Pl/CSF	RTV (ng/ml) Pl/CSF
0161	<40/1350	None detected	76/3.0	445/256	1490/4.4	3.0/3.0	3.0/3.0
0213	<40/1330	V82A/D67N/K70E/L74I/V106M/M184V/G190A	225/4.9	420/153	3.0/3.0	7730/7.0	935/3.0
0189	12802/310	K65R/V75I/M184V/L100I/K103N/P225H	82/3.0	146/175	1990/9.6	3.0/3.0	3.0/3.0
0133	19551/28250	M46I/V82A/D67N/K70R/M184V/T215F/K103N/K238T	19/5.0	63/71	3.0/3.0	14/3.0	3.0/3.0
0183	58000/1300	D67N/M184V/K103N/V106M	55/3.0	229/129	2460/3.0	3.0/3.0	3.0/3.0
0140	254/21370	K101E	33/3.0	98/123	5530/40	3.0/3.0	3.0/3.0
0164	620/2900	K101E	43/3.0	518/169	4130/8.8	3.0/3.0	3.0/3.0
0168	936/3030	None detected	56/3.0	286/196	2370/3.5	3.0/3.0	3.0/3.0
0172	24000/801	None detected	3.0/3.0	3.0/3.0	3.8/3.0	3.0/3.0	3.0/3.0

PID: participant ID. Pl: plasma. LPV: lopinavir. RTV: ritonavir. Limit of detection 40 copies/mL for VL and 3.0 ng/mL for antiretroviral drugs.

One participant (PID 0172) showed no detectable ART and, as expected, showed no CSF DRMs. HIV from one of two participants with CSF escape also contained DRMs (PID 0213), while HIV sequenced from the second participant (PID 0161) did not. The CSF escape participant with detected CSF DRMs was on a lopinavir/ritonavir protease inhibitor based therapy as detected by LC-MS/MS. DRMs included M184V, V106M, and G190A, the latter mutations a possible result of first line EFV regimen failure. In contrast, PID 0161, who was on EFV based therapy, had no CSF DRMs detected. Two additional participants (PID 0148 and 0164) showed CSF discordance, defined as a CSF VL about 0.5 *log*_10_ or greater of plasma VL [11]. Both were on EFV based therapy. EFV and FTC CSF concentrations in these participants were at or above the median CSF concentrations for suppressed and CSF escape participants (Table 2). Yet only the K101E DRM, conferring low level resistance to EFV (https://hivdb.stanford.edu/) was detected. An additional participant (PID 0168) on an EFV based regimen showed a CSF viral load. The level of EFV in the CSF was near background for this participant. However, FTC was above the median concentration for suppressed and CSF escape participants. No DRMS were detected in CSF virus from this participant. These results may indicate that DRMs may not be necessary for a detectable VL in the CSF in the face of ART.

## Discussion

Here, we examined the cellular source of viremia in study participants with CSF escape in Durban, South Africa, and whether drug levels in the CSF are lower in individuals with CSF escape versus those suppressed both in the blood and CSF. Detection of the CD26 T cell host surface marker on the viral envelope was consistent with the presence of at least some T cell origin virus in our study population, where the overwhelming majority of people living with HIV are infected with clade C virus [62]. Our conclusion that CD26 was effective in differentiating T cells from myeloid lineage cells was based on the observation that neither monocyte derived macrophages, nor primary human macrophages from human lymph nodes, nor human microglia, expressed CD26. Since only a fraction of T cells were positive for CD26, cells that were negative for CD26 could either be T cells or macrophages or microglia. However, expression of CD26 was only on T cells.

Consistent with this, HIV produced from *in vitro* infection of macrophages showed no CD26 expression. Given that CD26 is absent in myeloid lineage cells, a reasonable conclusion is that these cells did not generate virus with the CD26 host marker on its surface. This does not exclude the presence of virus produced in myeloid lineage cells in CSF escape, since about three-quarters of virions in the CSF did not express CD26 (S4 Fig), either because it was produced in myeloid lineage cells or because it was produced in T cells which did not express CD26.

Our results support the previously documented low levels of CD26 expression on macrophage origin HIV [46]. We did not find that CD36 was a robust marker to differentiate T cells from macrophages in our study population. We detected appreciable levels of CD36 on T cells, consistent with a recent study [63] but not two others [46, 64]. This was consistent with our *in vitro* infection results of T cells and MDM: Both T cell and MDM produced virus expressed CD36 at similar levels but MDM generated virus could be differentiated from T cell generated virus by the absence of CD26. Reasons for differences could include the specific study population, given the role of CD36 in fatty acid uptake [65]. T cell and macrophage surface marker detection in our study population was therefore critical in the choice of which marker to analyze.

To our knowledge, this is the first time CSF escape has been examined in Sub-Saharan Africa in a relatively large number of study participants. The CSF escape detected in this study was likely neurosymptomatic [59], and asymptomatic CSF escape may have a different mechanism of cellular persistence. The fraction of study participants with CSF escape, at 28%, was high relative to that found in other studies [11–16, 25]. This may be partly explained by the presence of co-infections and the selection of neurosymptomatic cases but may also be related to the HIV clade C subtype.

Infections are the most common cause of pleocytosis, the elevation of white blood cell numbers in the CSF [66]. Pleocytosis occurred in about half of participants with CSF escape, although the frequency was not significantly different from the suppressed group. Therefore, HIV could be produced in other cell types such as macrophages or microglia, then be amplified by the CD4+ T cells in the CSF. Our data does not exclude this possibility, it only indicates that some of the virions have a T cell origin. The data was also consistent with T cells producing HIV in the CSF in viremic individuals.

We did not observe lower ART levels in the CSF of CSF escape participants. CSF virus from two successfully sequenced CSF escape participants in our study showed DRMs in the participant on a protease based ART regimen, as determined by LC-MS/MS, but not in the participant on the first line EFV based regimen. Two additional participants on EFV based therapy showed CSF discordance, defined as a CSF VL about 0.5 *log*_10_ or greater of plasma VL [11]. CSF virus from both showed only low level resistance to EFV. With the limitation that the number of sequenced viruses was small, this may indicate that DRMs are not always essential for CSF escape or CSF discordance if the regimen is based on EFV. CSF escape despite the maintenance of suppressive ART and lack of drug resistance may be consistent with reactivation from latency [67, 68], more efficient HIV replication due to HIV cell-to-cell spread [58, 69–71], or both.

T cell origin HIV in CSF escape has been described in a recent study where two out of three participants with asymptomatic CSF escape had T cell tropic virus which was unlikely to utilize the low levels of CD4 expression found on macrophages [25]. This study was performed in a different population from that described in our study, and used techniques complimentary ours to arrive at similar conclusions. Infected T cells may therefore be present in the CSF in the face of ART, including in the Sub-Saharan African population most affected by HIV infection.

The observation that some T cells are infected and produce virus in the CSF is unexpected and may have important implications. Mechanisms driving such infections can include T cell infection by CNS resident cell types such as macrophages, ongoing infection between CNS resident CD4 T cells, or trafficking of infected T cells into the CSF from other compartments and potentially subsequent reactivation from latency. Unlike microglia and perivascular macrophages, which are compartmentalized to the CNS, T cells may circulate from and to other compartments. In addition, T cells may form a latent reservoir [72–77]. Given that potential curative approaches would likely need to eradicate all reservoirs [67, 68], the cellular nature of the CNS reservoir must be determined for these approaches to succeed.

## Supporting information

S1 Fig Gating strategy. (A) Gating for PBMC T cells (left two panels) and MDM (right two panels). Cells were stained with APC conjugated anti-CD3 (PBMC) or anti-CD68 (MDM) antibodies. (B) Gating for lymph node T cells. (C) Gating for lymph node macrophages. Representative result from one of 3 LN donors (024–09-0257).
(TIF)

S2 Fig No HIV infection of PBMC origin CD14+ cells. PBMC were infected with 2 × 10^7^ RNA copies/mL YFP-NL4–3(AD8). 2 days post-infection, cells were collected and stained with CD3 and CD14 antibodies, then analyzed for infection by detection of YFP positive cells in the CD3+ and CD14+ populations using flow cytometry. Shown are median and IQR for different blood donors. The median fraction of infected CD3+ gated CD4+ PBMC was 2.4% (IQR 1.7–3.2). No infected CD14+ monocytes were detected. The difference was significant (p-value is ****< 0.0001; Mann-Whitney U test). Brown circles denote blood donor 0020, red circles donor 0019, green donor 0049, and blue donor 0051.
(TIF)

S3 Fig Absolute HIV RNA copies from CD26 versus CD36 columns in *in vitro* infection. MDM or PBMCs were infected with NL4–3(AD8) macrophage tropic HIV able to infect both cell types. Supernatant from the infected cells was collected and diluted to 10^4^ HIV RNA copies/mL. Half the diluted supernatant was applied to CD26 binding columns and the other half to CD36 binding columns to quantify the number of CD26 and CD36 expressing virions. Shown are median and IQR for different blood donors. Macrophage values: CD26 median 208 HIV RNA copies/mL (85–440 copies/mL), CD36 median 5403 copies/mL (3076–9263 copies/mL). PBMC values: CD26 median 6600 copies/mL (5749–11185 copies/mL), CD36 median 5862 copies/mL (3309–5862 copies/mL). p-values are: *< 0.05; **< 0.01; ****< 0.0001 by Kruskal-Wallis non-parametric test with Dunn multiple comparisons correction.
(TIF)

S4 Fig Absolute HIV RNA copies per milliliter of CD26 expressing versus total virus in CSF escape samples. A viral load assay was performed on the CSF escape samples. Samples were then added to a column with anti-CD26 bead bound antibodies for immuno-capture of virus expressing CD26. Captured virus was then eluted and viral load assay performed. Red dotted line represents limit of assay detection. GM: geometric mean of n = 10 participants for each condition. Geometric mean was 5874 HIV RNA copies/mL for total virus, and 1591 copies/mL for virus with CD26 surface expression.
(TIF)

S1 Table Participant indications for lumbar puncture. (XLSX)
