# Supplementary figures and images for: T cell derived HIV-1 is present in the CSF in the face of suppressive antiretroviral therapy
Source: PLoS Pathog. 2021 Sep 23;17(9):e1009871. doi: 10.1371/journal.ppat.1009871 (PMC8509856; doi:10.1371/journal.ppat.1009871)

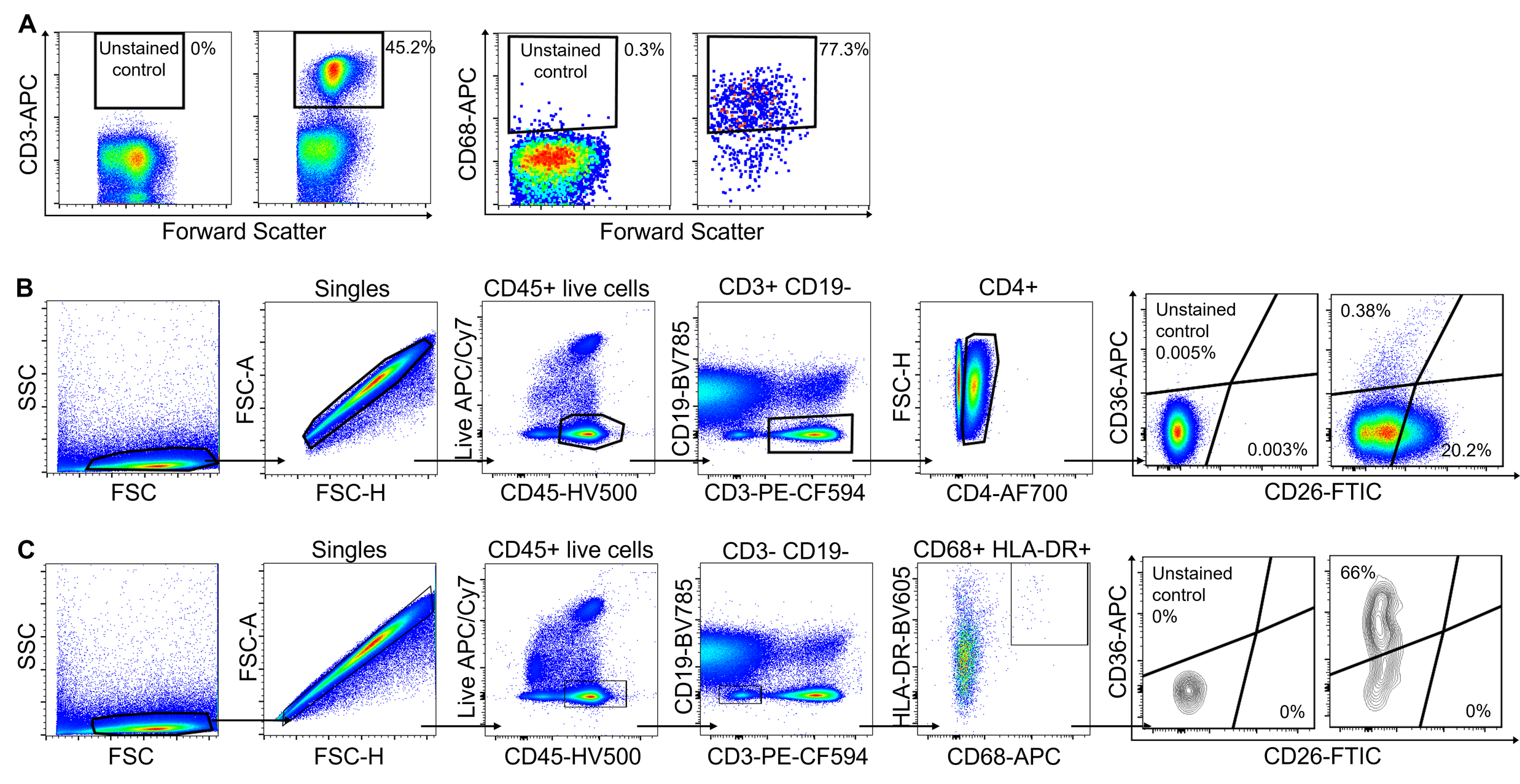

Supplement: S1 Fig — (A) Gating for PBMC T cells (left two panels) and MDM (right two panels). Cells were stained with APC conjugated anti-CD3 (PBMC) or anti-CD68 (MDM) antibodies. (B) Gating for lymph node T cells. (C) Gating for lymph node macrophages. Representative result from one of 3 LN donors (024–09-0257). (TIF) [file ppat.1009871.s001.tif]

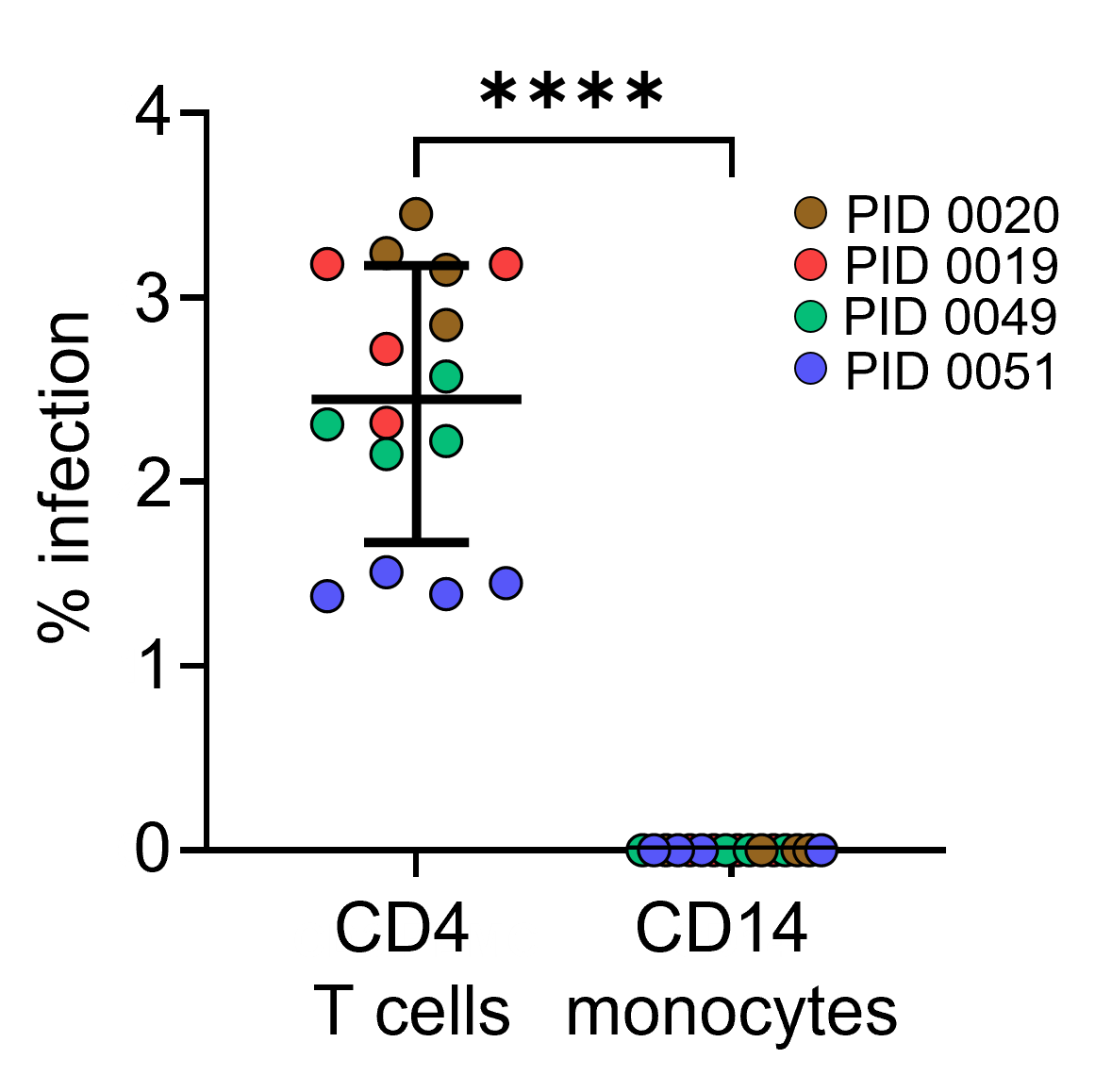

Supplement: S2 Fig — PBMC were infected with 2 × 10^7^ RNA copies/mL YFP-NL4–3(AD8). 2 days post-infection, cells were collected and stained with CD3 and CD14 antibodies, then analyzed for infection by detection of YFP positive cells in the CD3+ and CD14+ populations using flow cytometry. Shown are median and IQR for different blood donors. The median fraction of infected CD3+ gated CD4+ PBMC was 2.4% (IQR 1.7–3.2). No infected CD14+ monocytes were detected. The difference was significant (p-value is ****< 0.0001; Mann-Whitney U test). Brown circles denote blood donor 0020, red circles donor 0019, green donor 0049, and blue donor 0051. (TIF) [file ppat.1009871.s002.tif]

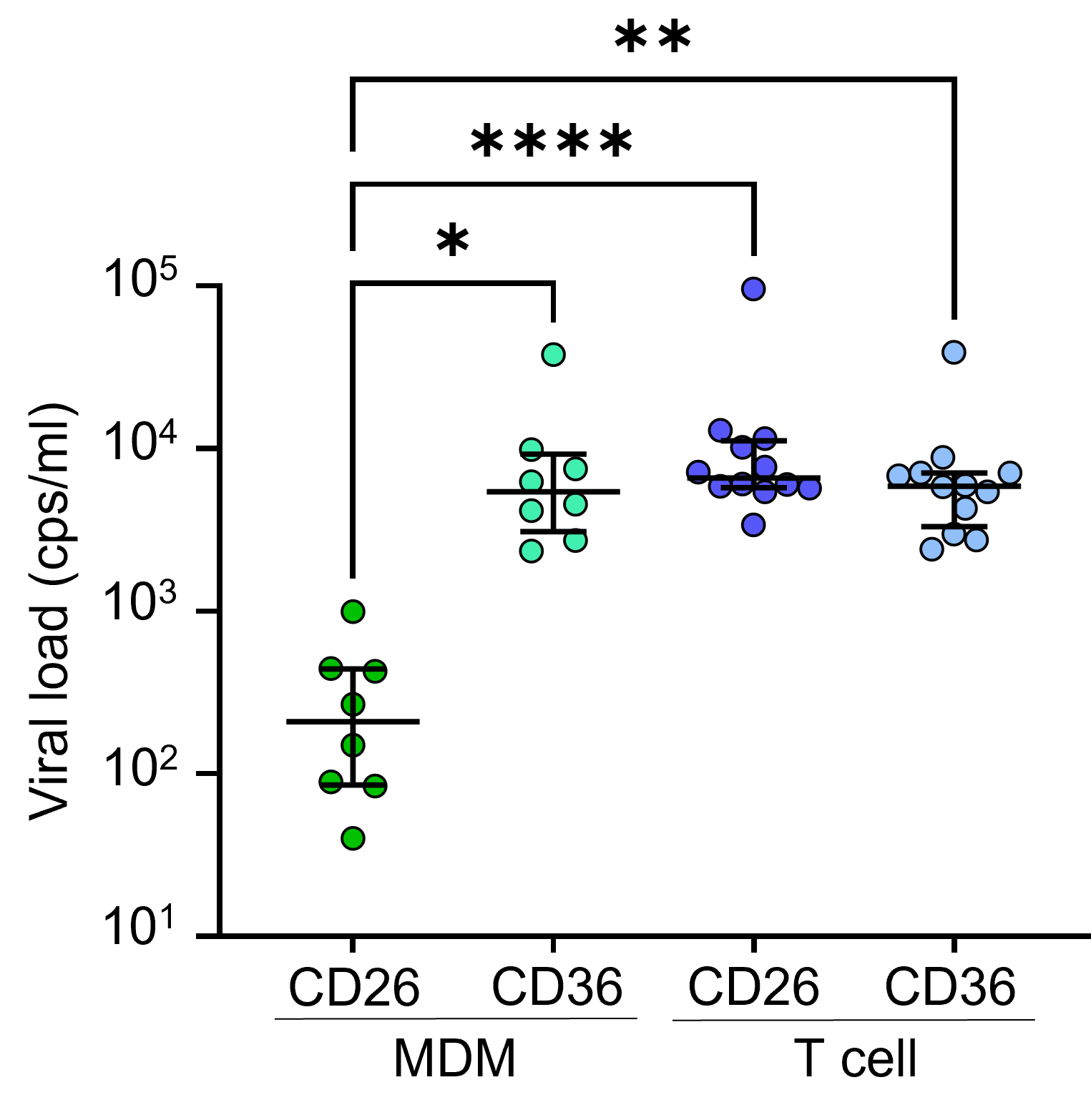

Supplement: S3 Fig — MDM or PBMCs were infected with NL4–3(AD8) macrophage tropic HIV able to infect both cell types. Supernatant from the infected cells was collected and diluted to 104 HIV RNA copies/mL. Half the diluted supernatant was applied to CD26 binding columns and the other half to CD36 binding columns to quantify the number of CD26 and CD36 expressing virions. Shown are median and IQR for different blood donors. Macrophage values: CD26 median 208 HIV RNA copies/mL (85–440 copies/mL), CD36 median 5403 copies/mL (3076–9263 copies/mL). PBMC values: CD26 median 6600 copies/mL (5749–11185 copies/mL), CD36 median 5862 copies/mL (3309–5862 copies/mL). p-values are: *< 0.05; **< 0.01; ****< 0.0001 by Kruskal-Wallis non-parametric test with Dunn multiple comparisons correction. (TIF) [file ppat.1009871.s003.tif]

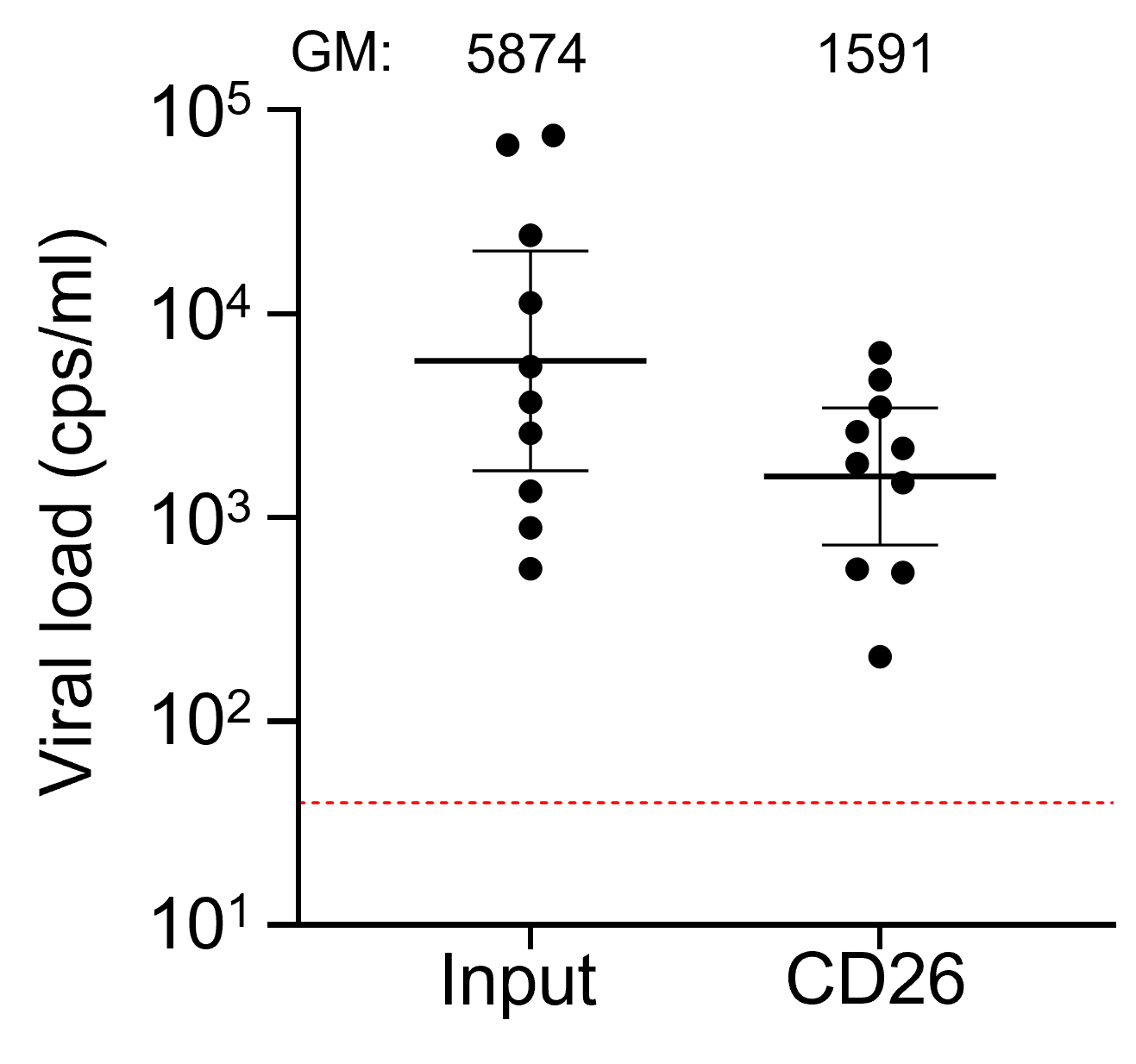

Supplement: S4 Fig — A viral load assay was performed on the CSF escape samples. Samples were then added to a column with anti-CD26 bead bound antibodies for immuno-capture of virus expressing CD26. Captured virus was then eluted and viral load assay performed. Red dotted line represents limit of assay detection. GM: geometric mean of n = 10 participants for each condition. Geometric mean was 5874 HIV RNA copies/mL for total virus, and 1591 copies/mL for virus with CD26 surface expression. (TIF) [file ppat.1009871.s004.tif]
